# Proteins of *Bartonella bacilliformis*: Candidates for Vaccine Development

**DOI:** 10.1155/2015/702784

**Published:** 2015-08-30

**Authors:** Cesar Henriquez-Camacho, Palmira Ventosilla, Michael F. Minnick, Joaquim Ruiz, Ciro Maguiña

**Affiliations:** ^1^Hospital Universitario Fundacion Alcorcon, Calle Budapest 1, 28922 Madrid, Spain; ^2^Instituto de Medicina Tropical “Alexander von Humboldt”, Universidad Peruana Cayetano Heredia, Lima 31, Peru; ^3^Division of Biological Sciences, University of Montana, Missoula, MT 59812, USA; ^4^ISGlobal, Barcelona Ctr. Int. Health Res. (CRESIB), Hospital Clínic, Universitat de Barcelona, 08036 Barcelona, Spain

## Abstract

*Bartonella bacilliformis* is the etiologic agent of Carrión's disease or Oroya fever. *B. bacilliformis* infection represents an interesting model of human host specificity. The notable differences in clinical presentations of Carrión's disease suggest complex adaptations by the bacterium to the human host, with the overall objectives of persistence, maintenance of a reservoir state for vectorial transmission, and immune evasion. These events include a multitude of biochemical and genetic mechanisms involving both bacterial and host proteins. This review focuses on proteins involved in interactions between *B. bacilliformis* and the human host. Some of them (e.g., flagellin, Brps, IalB, FtsZ, Hbp/Pap31, and other outer membrane proteins) are potential protein antigen candidates for a synthetic vaccine.

## 1. Introduction

*Bartonella bacilliformis* is a member of the alpha-2 subgroup of Proteobacteria and is the etiologic agent of Carrión's disease or Oroya fever in humans. Bartonellosis has been historically described in Peru, Ecuador, and Colombia [1, 2], with Peru considered the most important endemic area in the world. This disease often, but not always, presents in a biphasic manner. The primary, life-threatening acute phase is characterized by a septic state worsened by a severe hemolytic anemia (bacteria invade the erythrocytes which are destroyed by the spleen) [3]. This phase, known as Oroya fever, has a high fatality rate (up to 88%) if not treated and is more common in children [4]. Fever, pallor, general malaise, myalgia, headache, jaundice, and hepato/splenomegaly are the main symptoms of Oroya fever [1]. Some patients develop, as a consequence of the septic state, a transient immunosuppression that results in the onset of opportunistic infections such as toxoplasmosis, tuberculosis, salmonellosis, shigellosis, histoplasmosis, malaria, and pneumocystosis [1, 5, 6]. In the secondary chronic phase, known as verruga peruana (Peruvian wart), the bacteria invade the endothelial cells producing wart-like hemangiomatous lesions of the skin and mucous membranes 4 to 8 weeks after the onset of Oroya fever. The duration of the eruptive phase is 3 to 6 months. Lesions are classified as miliary (small reddish papules < 3 mm in diameter), mular (nodular tumors > 5 mm), and diffuse subdermal nodules [7]. Inhabitants, especially schoolchildren, in endemic regions often develop the eruptive phase as the sole manifestation of the disease [8]. Humans are the only known natural reservoir for* B*.* bacilliformis* [7, 9].* B*.* bacilliformis* is believed to be transmitted to humans by bites of phlebotomine sand flies living in the high mountain valleys of the Andes [10].

Since* B*.* bacilliformis* was first described in 1905 by Alberto Barton; numerous research studies have been reported, including clinical and experimental studies. In the last 25 years, arguably the most important studies have been conducted to explain the physiopathology of the disease.

The notable differences in clinical presentations during a* B*.* bacilliformis* infection suggest a complex adaptation to the human host with the objectives of persistence, maintenance of a reservoir state for vector-based transmission, and immune evasion. The bacterium-host relationship evokes a multitude of biochemical and genetic mechanisms involving both bacterial and host proteins.

**Figure 1:**
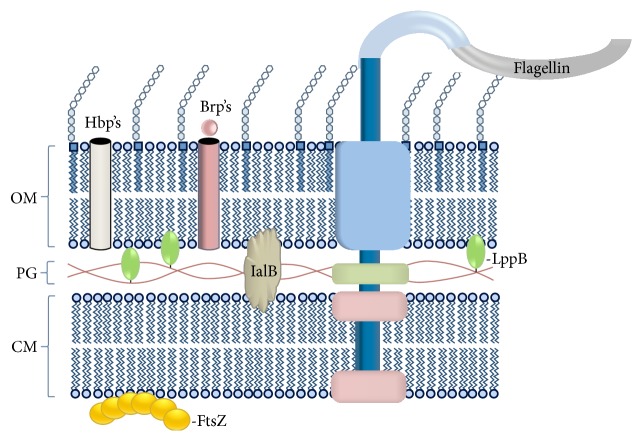
Various protein antigens of* B*.* bacilliformis* and their locations in the cell. Brp's:* Bartonella* repeat proteins, FtsZ: cell division protein, Hbp's: hemin-binding proteins, IalB: invasion-associated locus B protein, LppB: lipoprotein B, OM: outer membrane, PG: peptidoglycan, and CM: cytosolic membrane.

This review focuses on proteins involved in* B*.* bacilliformis*' interactions with the human host. We hypothesize that certain proteins would be ideal protein antigen candidates to be included in a chemically synthesized vaccine to block* B*.* bacilliformis* interactions with host cells (see Figure 1).

*Flagellin*.* B. bacilliformis* cells are highly motile by lophotrichous flagella, appendages consisting of ~42 kDa flagellin protein subunits that are resistant to protease or trypsin treatment [11].* B*.* bacilliformis* was reported to possess 1 to 10 polar flagella 3 to 10 *μ*m in length [11]. In addition to blood flow within the circulatory system, flagella provide the bacterium with a high degree of mobility and may serve to propel the pathogen during its search for erythrocytes [12].

Bacterial motility seems to be required for internalization, since entry of nonmotile* B. bacilliformis* was not observed in the experiments of Mernaugh and Ihler [13].

Antiserum to flagella significantly reduced* B*.* bacilliformis* association with red cells as compared to controls [11], and there was poor adherence of nonmotile variants and flagellin-minus (bald) mutants [14, 15]. These data suggest that flagella may possess adhesive qualities and/or they increase bacteria-host cell interactions [11]. In view of the significant decrease in erythrocyte infection due to antiflagellin antibodies, this protein is a promising antigen for use in a potential subunit vaccine. 

*Brps*.* Bartonella* species invade a variety of eukaryotic cells by employing trimeric autotransporter adhesion proteins (TAAs) [16]. The TAAs all use a type V secretion pathway and consist of a passenger domain and a beta domain used to deliver the passenger component out of the cell via the outer membrane.* B*.* bacilliformis* possesses three* brp* genes encoding potential* Bartonella* repeat proteins (Brps) [17].* B*.* bacilliformis* Brp proteins share common domains and structural features with the TAAs. While the biological role of* B*.* bacilliformis* Brp proteins has not yet been investigated, they may be involved in similar biological processes as the TAA proteins of* B*.* henselae* and* B*.* quintana*, including autoagglutination, adhesion to host cells and extracellular matrix proteins (fibronectin and collagens), inhibition of phagocytosis, and induction of a proangiogenic response in host cells [10]. Immune-mediated inhibition of these hypothetical functions could possibly confer protection against the pathogen. 

**Figure 2:**
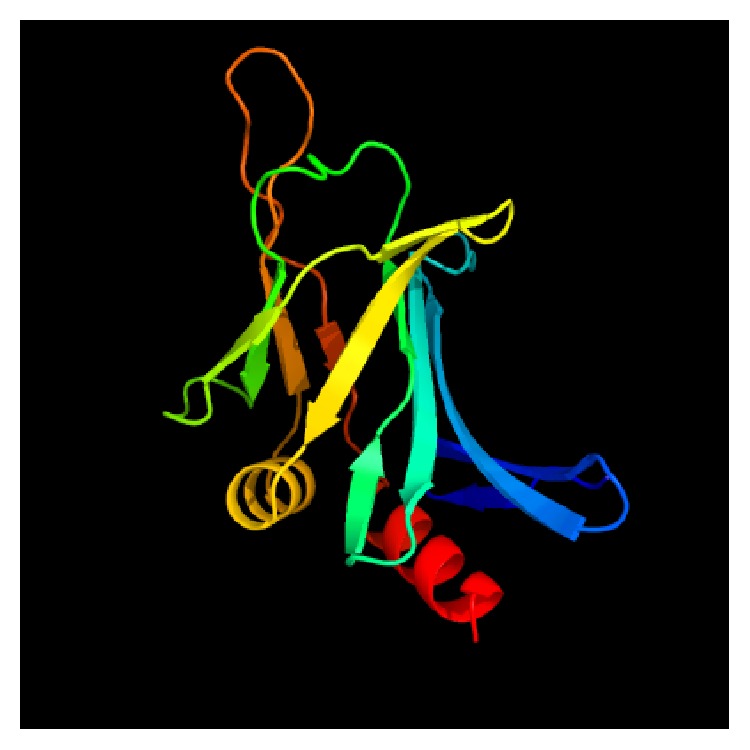
Crystal structure of the invasion-associated locus B protein; 144 residues (77% of* Bartonella bacilliformis* IalB sequence) modeled with 100.0% confidence by the single highest scoring template. Program Phyre2 at http://www.sbg.bio.ic.ac.uk/phyre2/html/page.cgi?id=index. Model based on* B*.* henselae* IalB crystal template c3dtdi_.

*IalB*. The invasion-associated locus B (IalB) protein (see Figure 2) was originally discovered by screening a* B*.* bacilliformis* genomic library for erythrocyte-invasive strains of host* E*.* coli* [18]. IalB has a significant effect on enhancing* B*.* bacilliformis*' erythrocyte invasion. It is possible that environmental changes during passage of the bacterium from sand fly to humans (e.g., changes in temperature, iron availability, and pH) have an effect on* ialB* gene expression [19]. In other research, IalB protein levels were correlated with changes in* ialB* mRNA and changes of temperature/pH of the growth media. The greatest amount of* ialB* was present in* B*.* bacilliformis* that had been acid-shocked (pH 5), grown at 20°C, or temperature downshifted to 20°C from 30°C. As with* ialB* mRNA levels, IalB protein amounts in* B*.* bacilliformis* decreased in response to basic pH (pH 8.0) or a temperature upshift to 37°C [20].

Presumably,* B*.* bacilliformis* undergoes a temperature shift from 37°C to 20°C when it is ingested by a sand fly, as the blood meal in the insect cools to ambient temperature. Concurrent with this temperature downshift, bacterial* ialB* expression would presumably be upregulated for erythrocyte invasion following transfer to another human host when the insect subsequently feeds. A change of the pH also occurs in the sand fly midgut (pH increases slightly to pH 7.4), where* ialB* expression would presumably be upregulated. When bacteria pass from the sand fly vector to a human host, the event would be signalled by a rapid upshift in temperature to 37°C and an increase in pH to 7.4. The* ialB* gene expression would be downregulated but not abrogated under these conditions. Undoubtedly, other bacterial factors are involved in erythrocyte adherence and invasion. IalB was localized to the inner membrane of* B*.* bacilliformis*, where it is unlikely to directly interact with erythrocytes. In contrast, the IalB homolog of* B*.* henselae* was localized to both inner and outer membrane fractions of the bacterium [21]. It is unknown if this protein is a transporter or signal transducer involved in human erythrocyte invasion, and its mechanism of action remains elusive [20]. 

*FtsZ*. The FtsZ protein of* B*.* bacilliformis* (FtsZBb) is a ~75 kDa protein and a structural homolog of* B*.* henselae*'s cell division protein, FtsZ [20]. FtsZBb is strongly immunogenic in humans and was one of two major antigens (i.e., 75 kDa and 65 kDa proteins) identified in early work [9, 22]. In addition, the C terminus of FtsZBb contains a 256-amino acid region that is unusual and found in other related bacteria such as* Rhizobium meliloti*. As a result, the FtsZBb is almost twice as large as the majority of the FtsZ homologs, perhaps contributing to its antigenicity [17]. Interestingly, the* ftsZ* gene has been included in MLST schemes to study clonal and evolutionary relatedness of* B*.* bacilliformis* strains [23].

## 2. Miscellaneous Outer Membrane Proteins

Several* B*.* bacilliformis* outer membrane proteins have been identified by a variety of means. Minnick identified 14 proteins ranging from 11.2 to 75.3 kDa. Three of these proteins (31.5, 42, and 45 kDa) were prominent immunoprecipitants when they were exposed to rabbit hyperimmune serum [24]. Iwaki-Egawa and Ihler identified six* B*.* bacilliformis* proteins that mediate bacterial interactions with erythrocytes (100, 92, 84, 46, 37, and 12 kDa) [25].

A 43 kDa immunogenic lipoprotein of* B*.* bacilliformis* was identified through screening of a genomic DNA lambda library. The protein is a homolog to the LppB proteins of* Haemophilus* spp. and the NlpD protein of* E*.* coli* and was thus designated LppB [24]. The LppB lipoprotein is likely to use the same biosynthetic pathway as other bacterial lipoproteins. The protein's prominent immunogenicity was demonstrated using five convalescent sera from patients with Oroya fever or verruga peruana [26].

Buckles and McGinnis Hill demonstrated that* B*.* bacilliformis* was able to bind to several human erythrocyte proteins, including *α* and *β* subunits of spectrin, band 3 protein, glycophorin A, and glycophorin B [27]. Band 3 is a major transmembrane glycoprotein of the erythrocyte membrane, and it may be one of the possible erythrocyte receptors of* B*.* bacilliformis* [27]. In addition, band 3 has been suggested to be involved* Plasmodium* invasion of erythrocytes [28, 29].

## 3. Hemin-Binding Proteins (Hbp's)

The* B*.* bacilliformis* hemin-binding proteins (Hbp's) are homologs of the bacteriophage-associated, Pap31, protein of* Bartonella henselae* [30] and the five hemin-binding proteins of* B*.* quintana* [31, 32].* B*.* bacilliformis* possesses three, tandem* hbp*-encoding genes in its chromosome. In addition to serving as a receptor for hemin and associating with the phage coat, data from work with* B*.* henselae* suggest that these proteins may also serve as adhesins for fibronectin, heparin, and host cells such as endothelial cells [33]. The Hbp (Pap31) proteins are highly expressed antigens in growing cultures of* B*.* bacilliformis* and are immunologically dominant, making them ideal antigens for use in enzyme-linked immunosorbent assays (ELISAs) and in immunoblots. In fact, use of this single protein antigen permits the use of ELISA, Western blot methods, or rapid lateral flow assay for the diagnosis of a* B*.* bacilliformis* infection [34]. To what extent* B*.* bacilliformis* Hbp's are immunoreactive in humans is unknown. However, the HbpE homolog of* B*.* quintana* is a commonly recognized antigen in sera from trench fever patients [35]. This observation suggests that one or more* B*.* bacilliformis* Hbp's may also be seroreactive in humans and thus useful subunit vaccine candidates.

## 4. Conclusions

*B*.* bacilliformis* infection represents an interesting model of human host specificity by a bacterium. While we now know more about the biology of the bacterium and the pathogenesis of Carrión's disease, we still lack adequate knowledge to select specific protein candidates to generate a subunit vaccine formulation. Studies regarding comparative genomics and proteomics are sorely lacking, especially considering the significant impact that bartonelosis outbreaks represent for the affected populations in South America. Carrión's disease is an emerging infectious disease that is not amenable to eradication with antibiotics or pesticides. Thus, the most effective intervention strategy to improve public health will be the development of a vaccine. Advances in* B*.* bacilliformis* genomics and proteomics hold the promise a rational vaccine design in the near future [36].
